# An Organotypic Microcosm for the Pancreatic Tumor Microenvironment

**DOI:** 10.3390/cancers12040811

**Published:** 2020-03-28

**Authors:** Miranda Lin, Mei Gao, Prakash K. Pandalai, Michael J. Cavnar, Joseph Kim

**Affiliations:** Department of Surgery, University of Kentucky, Lexington, KY 40536, USA; miranda.lin@uky.edu (M.L.); mei.gao@uky.edu (M.G.); prakash.pandalai@uky.edu (P.K.P.); michael.cavnar@uky.edu (M.J.C.)

**Keywords:** pancreatic cancer, organoids, tumor microenvironment

## Abstract

Pancreatic duct adenocarcinoma (PDAC) is projected to become the second leading cause of cancer-related deaths in the next few years. Unfortunately, the development of novel therapies for PDAC has been challenged by a uniquely complex tumor microenvironment. The development of in vitro cancer organoids in recent years has demonstrated potential to increase therapies for patients with PDAC. Organoids have been established from PDAC murine and human tissues and they are representative of the primary tumor. Further, organoids have been shown beneficial in studies of molecular mechanisms and drug sensitivity testing. This review will cover the use of organoids to study PDAC development, invasiveness, and therapeutic resistance in the context of the tumor microenvironment, which is characterized by a dense desmoplastic reaction, hindered immune activity, and pro-tumor metabolic signaling. We describe investigations utilizing organoids to characterize the tumor microenvironment and also describe their limitations. Overall, organoids have great potential to serve as a versatile model of drug response and may be used to increase available therapies and improve survival for patients with PDAC.

## 1. Introduction

Pancreatic duct adenocarcinoma (PDAC) is a devastating disease with a 5-year survival in the US of just 10% and a projection to become one of the top leading causes of cancer-related deaths by 2030 [1,2]. Pancreatic cancer is characterized by rapid growth and invasiveness, and therapeutic targeting is complicated by multiple facets of the tumor microenvironment, including desmoplastic reaction, immunosuppression, and complex metabolic interactions [3,4,5]. The dense, surrounding stroma contains cells that produce growth factors to feed the tumor and block drug delivery [3]. The stroma also reduces cytotoxic T-cell infiltration to the tumor and suppresses T-cell activity that is present at the tumor [5]. Additionally, rapid metabolism of active drugs in the body decreases effective killing of pancreatic tumors [4]. Therefore, there is an unmet need for in vitro models of the tumor microenvironment to combat the aggressive nature of PDAC and increase therapeutic options.

Organoid culture has gained interest as a valuable in vitro model of molecular interactions and drug sensitivity testing [6]. Organoids are 3D models that have been created from human and murine normal and tumor tissues, and human-derived organoids have been created from biopsy specimens and bulk surgical tissues [6,7,8]. They maintain tumor architecture, genetic profiles, and epigenetic changes of the tissue of origin [9]. Further, organoids created from patient tumor tissues are useful for drug sensitivity testing as biobanks for novel drug development or as individualized models of personalized medicine [9]. They are more clinically relevant than traditional models of human cancers such as 2D cell cultures, which are immortalized, grown in monolayers, and fail to represent tumor heterogeneity and the surrounding microenvironment, including stromal and immune components [7,9,10]. Additionally, patient-derived xenografts, which mimic in vivo interactions, require 4–8 months to develop before sufficient material is generated for drug sensitivity testing [9,10,11]. Organoids are rapid growing and less costly than in vivo models; therefore, they present an attractive alternative for the rapid study of molecular interactions and drug response.

The methods for establishing PDAC organoids from biopsy specimens and surgical tissues have been described [6,7,8]. Once tumor tissues have been obtained, they are placed immediately in organoid media. The tissues are digested in collagenase, Dispase; and TrypLE Express if further digestion is needed [7,8]. Once digested and washed, the specimens are suspended in Matrigel and plated on pre-warmed culture plates. The organoids are overlaid with complete organoid medium containing growth factors and other supplements such as Wnt, R-Spondin, Noggin, epidermal growth factor, fibroblast growth factor 10, B_27_ supplement, N-acetylcysteine, Gastrin, Primocin, nicotinamide, and Y27632 [6,7]. Organoids begin to grow within 1–2 days and once 80% confluent, they are passaged and may be expanded into additional wells or frozen for later use [7,8].

**Figure 1:**
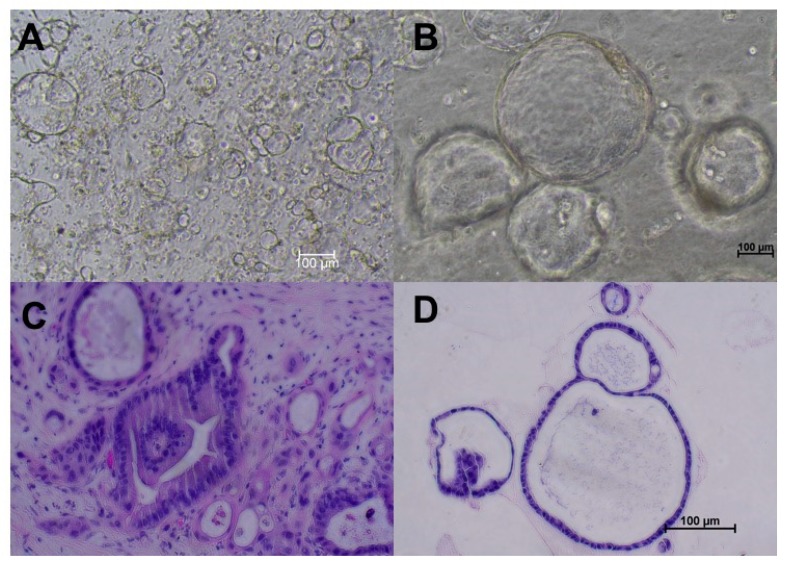
Pancreatic duct adenocarcinoma (PDAC) organoids created from specimens obtained from surgical resection from the same patient. (**A**) Passage 0 (P0) organoids on day 4 after creation. (**B**) P2 PDAC organoids. (**C**) Hematoxylin and eosin stain of tumor tissues from which organoids were derived (200×). (**D**) Hematoxylin and eosin staining of the same organoid line reveals nucleolar prominence and cell adhesions, confirming PDAC origin. All images were taken using the Nikon Ts2 microscope.

PDAC tumor organoids represent the morphologic features of the primary tumor (Figure 1). Compared to normal pancreatic organoids, tumor organoids contain nuclear irregularity, nucleolar prominence, and cell–cell adhesions as seen by hematoxylin and eosin staining [12,13]. Additionally, normal murine pancreatic organoids induced with a *Kras* mutation, which occurs in 94% of PDACs, displayed typical features of early PDAC, including multilayering epithelial cells, disorganized cells, and overlapping nuclei [14,15,16]. PDAC organoids also harbor pancreatic cancer markers CK7, CK19, P53, and lack of CK20, consistent with paired tumor tissues [12,13]. Furthermore, organoids are representative models of the genetic landscape of the tumor of origin and have been indicated as a beneficial drug sensitivity model for PDAC patients [17,18].

Organoids serve as a valuable model for translational cancer research. They are extremely versatile and may be manipulated to study important factors contributing to PDAC growth and invasiveness. Further, they are accurate models of drug sensitivity and some groups are currently incorporating organoids for testing drugs in human clinical trials (NCT03544255, NCT03500068). This review focuses on the use of PDAC organoids to study components of the tumor microenvironment important for PDAC survival, including stromal reinforcement, immune evasion, and the rapid metabolic breakdown of drugs.

## 2. Elucidating the Problem of Stromal Infiltration in Organoids

### 2.1. Stromal Interactions Lead to Increased Tumor Proliferation

A major challenge in the treatment of PDAC is the dense stroma surrounding pancreatic tumors. Desmoplasia, or the growth of fibrous connective tissue around the neoplasm, is a result of stromal cell infiltration and subsequent inflammation [3]. The desmoplastic reaction serves as a physical barrier to efficient drug delivery. Stromal cells nurture the tumor through signaling molecules, chemokines and growth factors, to promote growth and reduce tumor vasculature, which further inhibits efficient drug delivery [3]. Stromal cells in the extracellular matrix can make up to 80% of the tumor mass and include myofibroblasts, cancer-associated fibroblasts (CAFs), pancreatic stellate cells (PSCs), and myeloid-derived suppressor cells [5,19]. The production of signaling molecules by stromal cells attracts additional stromal cells to the tumor, resulting in a positive feedback loop that ultimately leads to more inflammation and exclusion of effector T-cells from the tumor. Many studies have investigated tumor–stroma interactions, some of which have elucidated methods that utilize mouse and human-derived PDAC organoids to overcome the dense network of stroma surrounding the tumor [12,20].

### 2.2. Culture of Mouse PDAC Organoids

Pancreatitis is a condition characterized by inflammation and fibrosis. Patients with chronic pancreatitis have a significantly increased likelihood of developing pancreatic intraepithelial neoplasia (PanIN) and subsequently, PDAC [10]. The sequence of tumorigenesis from normal phenotype to malignancy has been shown in organoids derived from murine tissues.

The glycan carbohydrate antigen 19-9 (CA19-9) is often elevated in serum and pancreas tissues of patients with chronic pancreatitis and PDAC. To study mechanisms involved in pancreatitis, Engle et al. induced transgenic mutations in mice to stimulate the production of CA19-9 [20]. Organoids subsequently created from CA19-9-expressing murine tissues retained the transgenic gene mutations and also produced CA19-9, highlighting the ability of organoids to represent the original genetic landscape and phenotypic consequences of tissues from which they are derived. Furthermore, conditioned media collected from CA19-9-expressing organoids stimulated epidermal growth factor receptor (EGFR) phosphorylation [20,21]. These data suggest a role for CA19-9 in EGFR-mediated induction of chronic pancreatitis. In addition, this study highlights the benefit of using organoids to study important biomarkers in pancreatic diseases.

Another important milestone in the progression from normal pancreatic tissue to pancreatitis is the loss of the protein, E-cadherin. The gene *Cdh1* encodes E-cadherin, which plays a role in cell-to-cell adhesion [22]. The role of this gene was studied extensively in murine-derived organoids by Kaneta and colleagues [22]. *Cdh1*-deficient organoids were collapsed, as expected from loss of cell adhesion, and showed signs of apoptosis, which morphologically appeared similar to pancreatitis. However, when *Kras*, one of the most commonly mutated genes in PDAC [15], was altered in organoids, *Cdh1*-induced apoptosis was reversed and organoids developed dysplastic, tall columnar cells [22]. These data suggest that combined *Cdh1* deficiency and *Kras* mutation promote progression to dysplasia and initiate tumor formation. Furthermore, organoid data paralleled in vivo studies revealing transformation to PanIN in nude mice after the induction of *Cdh1* loss and *Kras* alteration [22]. Additionally, organoids harboring *Cdh1* loss and *Kras* mutation were transplanted back into nude mice, which induced the growth of tumors that represented poorly differentiated adenocarcinoma in contrast to mice transplanted with organoids containing either mutation alone. This highlights the versatility of organoids in their ability to be transplanted into mouse models to induce fibrosis and tumor formation, and other studies have also demonstrated success transplanting PDAC primary and metastatic murine organoids into nude mice [7,10]. Overall, these studies demonstrate the utility of murine-derived organoids to elucidate the role of molecular changes inherent in the progression from normal pancreatic tissues to PanIN to PDAC.

### 2.3. The Role of Organoid Fibroblasts in Modeling Tumor–Stroma Interactions

A useful method for studying tumor–stroma interactions includes cell line or human tissue-derived organoids cocultured with commercial fibroblasts lines or fibroblasts isolated from patients [4,12,23,24,25,26]. Several publications have reported using cell line-derived organoids cultured with tumor-associated fibroblasts to study drug delivery systems and treatment resistance in pancreatic cancer [4,23,24,25]. Cell line-derived organoids cultured with fibroblasts had heterogenous distribution in size and shape. Importantly, fibroblasts in coculture subsequently condensed organoids into a larger solid mass, which exhibits the ability of stromal cells to contribute to the growth of PDAC tumors [4].

**Figure 2:**
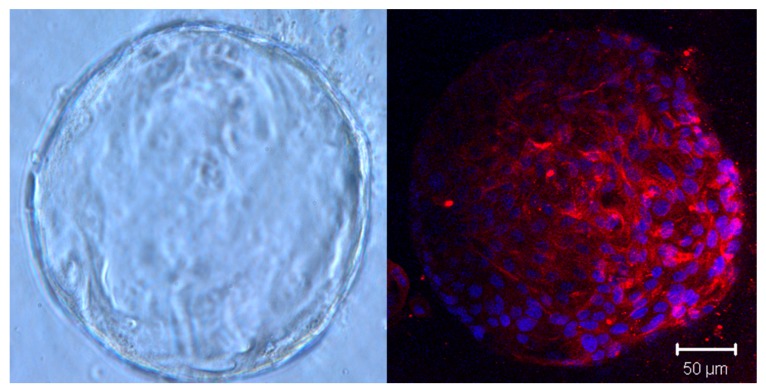
Brightfield image of a single P3 PDAC organoid (magnification, 200×) and expression of fibroblast-associated protein (red) in the same organoid line. DAPI was used for nuclear counterstaining and immunoflourescence was visualized with confocal microscopy (Zeiss 510 Meta NLO).

While cell lines are commercially available and can rapidly develop into organoids, they have limitations in their ability to accurately represent characteristics of the primary tumor. Therefore, studies have turned attention to human tissue-derived organoids obtained from surgery or biopsy [6,7,8]. Unlike cell line-derived organoids, patient-derived organoids contain a lumen, multiple cell types, and apicobasal polarity [12]. In addition, unlike cell lines, the successful growth of patient-derived PDAC organoids depends on specific growth factors important for tumor proliferation, which more accurately depicts niche dependency of the primary tumor [12]. PDAC organoids grown directly from patient tumor tissues may contain visible fibroblast growth at early (<P4) passages [12]. Fibroblasts attach to the well surface and express characteristic markers, such as fibroblast-associated protein (Figure 2) and vimentin [12]. Innate fibroblasts disappear with increased passaging; however, they are able to be isolated from organoids, expanded in 2D culture, and cocultured with new organoids, as demonstrated by Tsai et al. [12].

Patient-derived PDAC organoids have also been utilized to study basement membrane architecture and stromal invasion. Tubular ductal adenocarcinoma is the most common histopathological type of PDAC and is characterized by neoplastic cells forming ductal structures and invading the surrounding stroma [26]. Pancreatic epithelial cells are separated from the stroma by a basement membrane, which is highly cross-linked and insoluble. Destruction of the basement membrane is an important step in the invasion of tumor cells to the stroma.

Koikawa et al. developed the ability to model basement membrane architecture and observe factors contributing to basement membrane destruction and subsequent stromal invasion in PDAC organoids [26]. They established patient-derived PDAC organoid systems cocultured with human PSC lines, which play a role in basement membrane integrity, to model collagen matrix invasion. PDAC organoids were co-cultured with PSCs either in the same collagen matrix or separated by a permeable membrane. PDAC organoids in direct contact with PSCs demonstrated increased invasiveness, which was visualized using a collagen matrix migration assay and real-time imaging [26]. In response to direct contact of organoids with PSCs, organoid ductal structure was lost, basement membrane integrity declined, and there was observed migration into the collagen matrix compared to organoids separated from PSCs. In addition, using organoids, the researchers were able to confirm the role of matrix metalloproteinases, which are located on PSCs and break down extracellular matrix to clear the way for PDAC migration [26]. These data not only exhibit the ability of PDAC organoids to model basement membrane interactions, but also PSC-induced tumor invasion to the surrounding stroma, which has not been previously reported.

Cancer stem cells are dependent on a tumor microenvironment that secretes factors such as cytokines, Wnt, and Notch growth factors, to maintain stemness and promote self-renewal, angiogenesis, and metastasis [27]. Organoid technology has now led to our ability to model exogenous niche independency in various gastrointestinal cancers [28,29,30]. Seino et al. identified a functional PDAC organoid subtype that had lost reliance on exogenous Wnt signaling for survival and they hypothesized that surrounding stromal cells were a sufficient source of Wnt factors [30]. The group utilized a coculture system containing biopsy-derived PDAC organoids and attached patient-derived CAFs, which revealed organoid proliferation in the absence of exogenous Wnt. Dependence on Wnt-producing CAFs was also evident from failure of Wnt-deficient PDAC organoids without CAFs to successfully engraft in in vivo models [30]. These results are important, suggesting direct support of PDAC tumors from the surrounding stroma via niche signaling and further highlighting the utility of organoid coculture systems for studying tumor–stroma interactions.

### 2.4. Efficient Drug Delivery to Overcome High Fibrosis in the Pancreas

The stromal component of the tumor microenvironment presents a great challenge to the delivery of effective chemotherapies to the tumor. There are a few mechanisms of this blocking, including a physical barrier against drugs and secretion of intercellular signals to promote tumor survival [14]. Organoids cocultured with stromal cells have been used to study drug effects against PDAC cells. It should be noted that the activation status of fibroblasts (i.e., healthy vs. CAFs) has an effect on the degree of treatment response observed in organoids and should be taken into consideration when performing drug sensitivity studies [4]. The chemokine CXCL12, secreted by CAFs, has been shown to induce resistance to the chemotherapeutic agent gemcitabine in PDAC cell lines by inhibiting apoptosis [31]. Tsai et al. were able to model this decreased sensitivity to gemcitabine in PDAC organoids cultured with CAFs [12]. Importantly, the organoids created in this study were derived from human PDAC tissues and the patients’ own stromal cells, indicating potential for a personalized vehicle for drug screening for individual patients.

Organoid coculture systems have tremendous potential to serve as drug screening platforms for PDAC. Walsh et al. isolated fibroblasts from murine PDAC organoid cultures and tracked response to various drugs as single agents and in combination [32]. While the drugs failed to induce significant response in the fibroblasts, a novel platform of fibroblasts isolated from PDAC organoid cultures was developed that may be utilized for future stromal-targeting drug screens [32].

Moreover, coculture studies have been used to test novel photodynamic drug delivery systems [23,33]. One report utilizes heterotypic PDAC organoids created from MIAPaCa-2 cells cultured with patient-derived pancreatic CAFs as a model for testing a novel cetuximab photoimmunonanoconjugate delivery system [23]. Cetuximab is a monoclonal antibody that targets EGFR, which is overexpressed in many PDAC tumors and CAFs, and therefore may serve as a valuable target to eliminate the desmoplastic barrier [23,34,35,36]. The cetuximab photoimmunonanoconjugate efficiently penetrated PDAC organoid-fibroblast cultures after 1 hour of incubation [23]. Further, by inhibiting EGFR, which is necessary and sufficient for desmoplastic reaction [21], with cetuximab, this technique may provide an avenue of tumor penetration and destruction when coupled with chemotherapy. Together, these studies confirm the versatility of organoids to represent multiple cell types in vitro, and highlight the utility of organoids as a model for novel drug development.

## 3. Tracking Metabolic Transformations in PDAC Organoids

Metabolic activity in the PDAC microenvironment serves as an important signal between the tumor and its surrounding stroma. Metabolic interactions have been studied extensively in PDAC as a potential therapeutic target and have been modeled in organoids [4]. Broekgaarden and colleagues cocultured cell line-derived organoids with CAFs to study the metabolic impact of fibroblasts on the tumor and resulting therapeutic resistance [4]. PDAC organoids cultured with CAFs were resistant to oxaliplatin and photodynamic therapy compared to PDAC organoids alone. As expected, they found that PDAC organoids cultured with CAFs had increased redox states compared to PDAC organoids alone and the increased redox states in organoid-fibroblast cultures did correlate with the overexpression of oxidative stress pro-survival markers, including NF-κB and NRF2 [4,37]. The researchers elucidated a mechanism for this observation whereby CAFs decrease glutaminolysis in PDAC organoids, promoting a shift to oxidative phosphorylation, and therefore, chemotherapy and photodynamic therapy resistance [4]. These data fit with prior reports that indicate *Kras*-mutated PDACs rely heavily on oxidative phosphorylation [38]. Broekgaarden et al. proceeded to treat PDAC organoids cultured with CAFs with metformin, an inhibitor of mitochondrial complex I and oxidative phosphorylation, in combination with oxaliplatin and photodynamic therapy [4]. While the results were not significant, they did observe increased cytotoxicity from the addition of metformin to oxaliplatin and photodynamic therapy. These data further support the use of organoids to study metabolic states in PDAC tumors and their potential for serving as models of drug discovery.

An ongoing challenge in the development of cancer therapies is overcoming the rapid metabolism of chemotherapies by metabolic enzymes such as cytochrome P450. The cytochrome P450 proteins are a family of enzymes located in the endoplasmic reticulum of cells and are responsible for either the bioactivation or break down of drugs [39]. The metabolic effect of cytochrome P450 is drug-dependent and may be modeled in organoids. Park and colleagues established mouse liver organoids grown in either undifferentiated or differentiated media [39]. A subset of organoids grown in differentiated media was prepared for induction of cytochrome P450. All three liver organoid groups were then cocultured with PDAC organoids and treated with docetaxel. Pancreatic cancer organoids cocultured with liver organoids induced with cytochrome P450 survived, while those not cultured with P450 were killed. These data suggest cytochrome P450 metabolizes docetaxel into its inactive form and mediates drug resistance in PDAC. This study also further describes the utility of organoids for studying metabolism in the pancreatic tumor microenvironment.

Similarly, optical metabolic imaging (OMI) is a noninvasive method of measuring cellular metabolic state that has been applied in organoid technology [32]. Metabolic imaging is useful for measuring therapeutic efficacy because tumor size regression occurs secondary to metabolic effects [40]. Therefore, OMI provides the ability to identify early biomarkers of drug sensitivity or resistance. OMI measures the redox ratio and fluorescence lifetimes of the two metabolic cofactors, nicotinamide adenine dinucleotide (NADH) and flavin adenine dinucleotide (FAD), to yield an OMI index that indicates the metabolic output of cells. OMI has previously been used to successfully measure metabolism and drug response in human breast cancer cells and xenografts [40].

Walsh et al. used OMI to correlate the metabolic states of human PDAC organoids to drug response [32]. They treated organoids with the chemotherapeutic agent gemcitabine and the small molecule JAK inhibitor AZD1480 as single drugs and in combination. Their analyses revealed a significant reduction in the OMI index of PDAC organoids in response to gemcitabine and the combination of gemcitabine and AZD1480, which correlates to high cytotoxicity compared to controls and AZD1480 alone [32]. Murine PDAC organoids treated with single and combination therapies and analyzed by OMI also revealed metabolic changes corresponding to drug response. The observed metabolic changes also correlated with expression of proliferation and apoptosis markers in the same organoids [32].

Using OMI analysis in PDAC organoids, Walsh et al. were also able to track initial response and subsequent decreased sensitivity to AZD1480 in murine PDAC organoids over time [32]. Similar to the results described by Park et al. [39], these data support the ability of organoids to represent acquired therapeutic resistance. Additionally, organoids grown from murine biopsies in this study contained three different morphologies (primary tumor, metastatic lesions, and fibroblasts), varying basal metabolic states, and different coenzyme environments and binding affinities, which highlights the versatility of organoids as an accurate model of heterogeneity within the primary tumor and its surrounding environment.

## 4. Immune Modeling in PDAC Organoids

### 4.1. Targeting an Immunosuppressive Tumor Microenvironment

Therapeutic targeting in PDAC has also been limited by the inability to activate the body’s innate immune mechanisms. PDAC is considered to be a nonimmunogenic cancer due to numerous factors, including a lack of T-cell infiltration into the tumor and an immunosuppressive microenvironment [5].

Due to its low mutation burden compared to other cancer types, PDAC tumors have low neoantigen loads and therefore, a lack of tumor-infiltrating T-cells [5]. The dense surrounding stroma also precludes T-cell trafficking to the tumor. Typically, neoantigens, which result from a high mutation burden, are presented as non-self to circulating lymphocytes and subsequently activate an immune response. Immunotherapy, such as immune checkpoint inhibitors, enhance this response by targeting immunosuppressive immune checkpoints, programmed cell death protein 1 and cytotoxic T-lymphocyte-associated protein 4 [5]. Since there is little T-cell infiltration into PDAC tumors, immune checkpoint therapy is inadequate and has failed in human clinical trials [41,42].

### 4.2. Coculture of Immune Populations with PDAC Organoids

To combat the lack of T-cell infiltration into the tumor, studies have been investigating vaccines to induce CD8+ T-cells in PDAC [43]. The ability to increase the concentration of immune cells in PDAC tumors may complement immune checkpoint inhibition, and organoids provide a vehicle for these studies in vitro. Tsai et al. demonstrated successful isolation and growth of viable human CD4+ and CD8+ T-cells [12]. T-lymphocytes grown in culture with organoids infiltrated the matrigel boundary and migrated towards the organoids, as opposed to T-lymphocytes grown in coculture with empty matrigel domes [12]. Organoid cultures were able to display tumor-driven migration of immune cells into the tumor microenvironment and therefore, have the potential to investigate novel therapies combining immune vaccines with immune checkpoint inhibitors.

It has been demonstrated that even when immune cells are present in the tumor microenvironment, they are suppressed [5]. Myeloid-derived suppressor cells in PDAC inactivate antigen-specific T-cell immunity, and macrophages in the PDAC microenvironment are skewed towards a tumor-promoting phenotype [5,44]. Recent studies modeled macrophage transformation in the presence of PDAC organoids. Bishehsari et al. developed a platform using a transwell insert to coculture murine macrophages with either *Kras*-mutated or wild-type murine pancreatic organoids [14]. *Kras*-mutated organoids began to develop defining features of early PDAC compared to wild-type organoids. In culture with macrophages, *Kras*-mutated organoids induced a shift in macrophages to a pro-tumorigenic phenotype and the organoids exhibited acinar to ductal differentiation [14]. These data further emphasize the detrimental impact of PDAC cells on the body’s innate immunity and this relationship is portrayed in organoid cultures.

Bishehsari et al. further investigated the interactions between macrophages and pancreatic tumor cells using organoid technology [14]. Macrophages initially in culture with *Kras*-mutated organoids were subsequently reseeded with a new *Kras*-mutated organoid line, which promoted ductal transformation and increased proliferation of the organoids. Overall, macrophages that were transformed from an anti-tumorigenic to a pro-tumorigenic phenotype later promoted the growth of *Kras*-mutated organoids in fresh culture [14]. These studies elucidate a feed-forward relationship among epithelial organoids and macrophages, potentially via the EGF/EGFR axis, and importantly, describe a useful coculture system for studying tumor–macrophage interactions and the role of macrophages in the tumor microenvironment.

Pancreatic tumors uniquely use their surroundings to suppress innate immunity by inhibiting T-cell infiltration and actively turning off immune cells. This results in considerable resistance to immunotherapy, including immune checkpoint inhibition, which has demonstrated success in various other cancers [41,45,46], but not PDAC [41,42]. Organoid technology provides an avenue for studying tumor-immune interactions and has potential to serve as a valuable model for testing novel therapeutics combining immune activators with cytotoxic chemotherapies.

## 5. Biomimetic Organoid Culture

Recently, improvements in biotechnology have advanced traditional organoid culture techniques with the use of synthetic polymers to improve the uniformity and accuracy of organoids [47,48,49]. Here, we will briefly discuss the use of bioprinting and microfluidics in organoids to recapitulate the PDAC tumor and its microenvironment. The main types of bioprinting include inkjet printing, extrusion-based printing, laser-assisted printing, and stereolithography [47]. Hakobyan et al. used laser-assisted bioprinting to generate square arrays of pancreatic spheroids with high accuracy and control over organoid number [50]. The organoids successfully modeled initiating events in PDAC development, including EGFR translocation to the cell membrane and acinar-to-ductal transformation [50]. Bioprinting is especially beneficial for plating cells for drug sensitivity testing, as demonstrated by Hou et al. [25]. They first isolated and immortalized tumor, metastasis, and fibroblast cells from patients with PDAC. Then, NanoShuttle, a reagent containing gold, iron oxide nanoparticles, and poly-L-lysine, was added to the cells to magnetize them for bioprinting and spheroid induction on a magnetic drive [25]. With bioprinting, the researchers were able to plate spheroids in a 384-well plate and screen 114 different National Cancer Institute oncology drugs. When using bioprinting technology to study molecular interactions and drug sensitivity, it is important to consider the physiological stress, including physical forces and biological stressors, required for the bioprinting process [51]. Therefore, bioprinting may be more suitable for highly-proliferative, robust cell line-derived spheroids as opposed to slower growing patient-derived organoids [51].

Microfluidics is the utilization of small channels to deliver precise volumes of fluids that allows for controlled physical and chemical analyses, and this technology has been applied in the context of PDAC organoid culture [52,53]. Using a microchannel plate, Lee et al. cocultured PDAC cell line-derived organoids with PSCs [52]. The plate included three compartments, one for organoids and two for PSCs, surrounded by four media channels. This system provided ability to study paracrine activation by PSCs and subsequent growth in the number of organoids [52]. They also measured PSC-stimulated organoid migration, including the number of cells migrating, distance moved through the extracellular matrix, and expression of epithelial-mesenchymal transition markers [52]. While useful for detailed analyses and high-resolution imaging, microfluidic technology is limited in that organoid retrieval from microfluidic devices may be difficult, they can only hold a small number of organoids, and the polydimethylsiloxane matrix used to hold the organoids may adsorb small hydrophobic molecules, presenting a confounding variable for drug screening studies [48].

Both traditional organoid culture and microfluidics have system limitations. Traditional organoid culture is restricted in that there is little control over organoid size, and microfluidic technology is limited by the small organoid number and a matrix that may confound drug testing results. However, Zhou et al. combined both culture systems and designed a novel microfluidic system that purifies organoids, which are subsequently plated on a traditional culture plate [53]. The microfluidic device includes four inlets/outlets and a main channel that contains hundreds of organoid units. Each organoid unit is funnel shaped to isolate spheroids from single cells and debris, providing control over organoid size. The researchers isolated and generated mouse-derived PDAC organoids, filtered them through their microfluidic device, and then plated them on a 96-well plate at one spheroid per well and observed their growth [53]. Their assay yielded viable organoids that proliferated rapidly over two weeks, which is sufficient time for organoid propagation or further molecular or drug sensitivity analyses [53]. Further, the polymerase chain reaction of filtered organoids revealed mRNA expression of mutant *Kras*, *Emr1* (macrophage marker), and *αSMA* (fibroblast marker), suggesting multiple cell types from the tumor microenvironment were retained in culture [53]. Advances in biotechnology have greatly enhanced organoid culture and their ability to model tumor interactions.

## 6. Limitations

As an in vitro model, organoids are limited in their ability to represent angiogenesis and metastasis to distant organs on a culture plate. In addition, while organoids contain multiple cell types, it has been shown that over time, specific cell types will emerge as dominant in culture [9,23]. Further, organoids in culture are of varying sizes, making it difficult to accurately measure cell death. However, our group and others are currently developing techniques to create uniformly-sized organoid cultures (unpublished) [53]. Lastly, organoid lines are expensive to maintain compared to cell lines; however, they are less costly than xenografts, making them an appropriate model to use prior to in vivo studies [8].

## 7. Conclusions

PDAC is a deadly disease that has had dismal outcomes in human clinical trials [41,42]. The complex microenvironment surrounding pancreatic tumors presents a unique challenge in the development of effective therapies for patients with PDAC. Organoids may be successfully developed and propagated from pancreatic normal and tumor tissues to study molecular interactions and have been implicated to facilitate personalized selection of therapies in human trials [18]. Since numerous extra-tumoral factors contribute to pro-cancer signaling in PDAC, the implementation of combination therapies to suppress the desmoplastic reaction and enhance the body’s innate immunity may provide an efficacious solution for improving survival in PDAC patients. As novel therapeutics are developed, organoid culture enhanced by improvements in biotechnology provides a useful vehicle for studying microenvironment interactions, identifying new biomarkers, and facilitating drug sensitivity studies.
